# Framing Continental Shelf Waves in the southern Adriatic Sea, a further flushing factor beyond dense water cascading

**DOI:** 10.1038/s41598-017-18853-2

**Published:** 2018-01-12

**Authors:** Davide Bonaldo, Mirko Orlić, Sandro Carniel

**Affiliations:** 1 Institute of Marine Sciences, National Research Council (CNR-ISMAR), Venice, 30122 Italy; 2 Andrija Mohorovičić Geophysical Institute, Faculty of Science, University of Zagreb, Zagreb, HR-10000 Croatia

## Abstract

Continental Shelf Waves (CSWs) are oscillatory phenomena migrating along the continental margins, controlled by the interplay of rotation and bathymetric gradients. Here we combine observational data from five moored current meters and high-resolution hydrodynamic model fields for describing the generation and propagation of CSWs along the Southern Adriatic Margin (SAM, eastern Mediterranean Sea), where the possibility of their occurrence has been theoretically hypothesised but not experimentally observed up to now. Results show that in spring 2012 a train of CSWs with 35–87 km wavelength and 2–4 day period was generated on the northern sectors of the SAM and propagated southwards along its western slope. Along their path, CSWs modify their apparent frequency and oscillation mode as an effect of the background current and scattering caused by changes in the continental margin morphology. This signal appears as a persistent feature triggered by the inflow of a dense water vein formed in the northern Adriatic Sea, propagating upwelling and downwelling patterns along broad sectors of the continental slope. CSWs thus appear as an additional remote-controlled mechanism for cross-shelf exchange of water, sediment and nutrients in the SAM, besides the well-acknowledged dense water downflow along preferential pathways driven by local topographic constraints.

## Introduction

Topographic waves are a category of oscillatory features associated with a modulation in the potential vorticity field induced by topographic gradients into a quasi-geostrophic flow^1–3^ in the ocean. The spatial characteristics of topographic waves and their propagation dynamics strongly depend on the geometrical constraints and, in principle, on the stratification conditions of the system. This allows for a broad variety of wave types, all propagating with the coast on their right (left) side in the northern (southern) emisphere. Along with the development of a sound theoretical framework for such waves, their dynamics and implications for coastal dynamics were progressively explored by means of observational campaigns, generally relying on current meter arrays deployed as single or composite moorings^4,5^. This permitted to highlight, for instance, the role of topographic waves in modulating heat and momentum fluxes below the thermocline on the Gulf Stream^4,6^ and coastal upwelling along the Australian coasts^7^.

Following analytical and observational studies, numerical modelling efforts have been progressively pursued allowing a deeper characterization of topographic wave dynamics, depending on variable meteo-marine and geographical conditions. Thus, dedicated high-resolution model runs in idealized configurations allowed to characterise the generation and propagation of topographic waves on a continental shelf in response to a tropical cyclone^8^, or as a lee effect of headlands or submarine canyons^9^. A set of realistic numerical simulations in the Mediterranean Sea led to identify the possible existence of topographic waves (with 2–5 day period) in the Strait of Sicily in response to wind stress curl or interactions between currents and seabed topography^10^.

**Figure 1:**
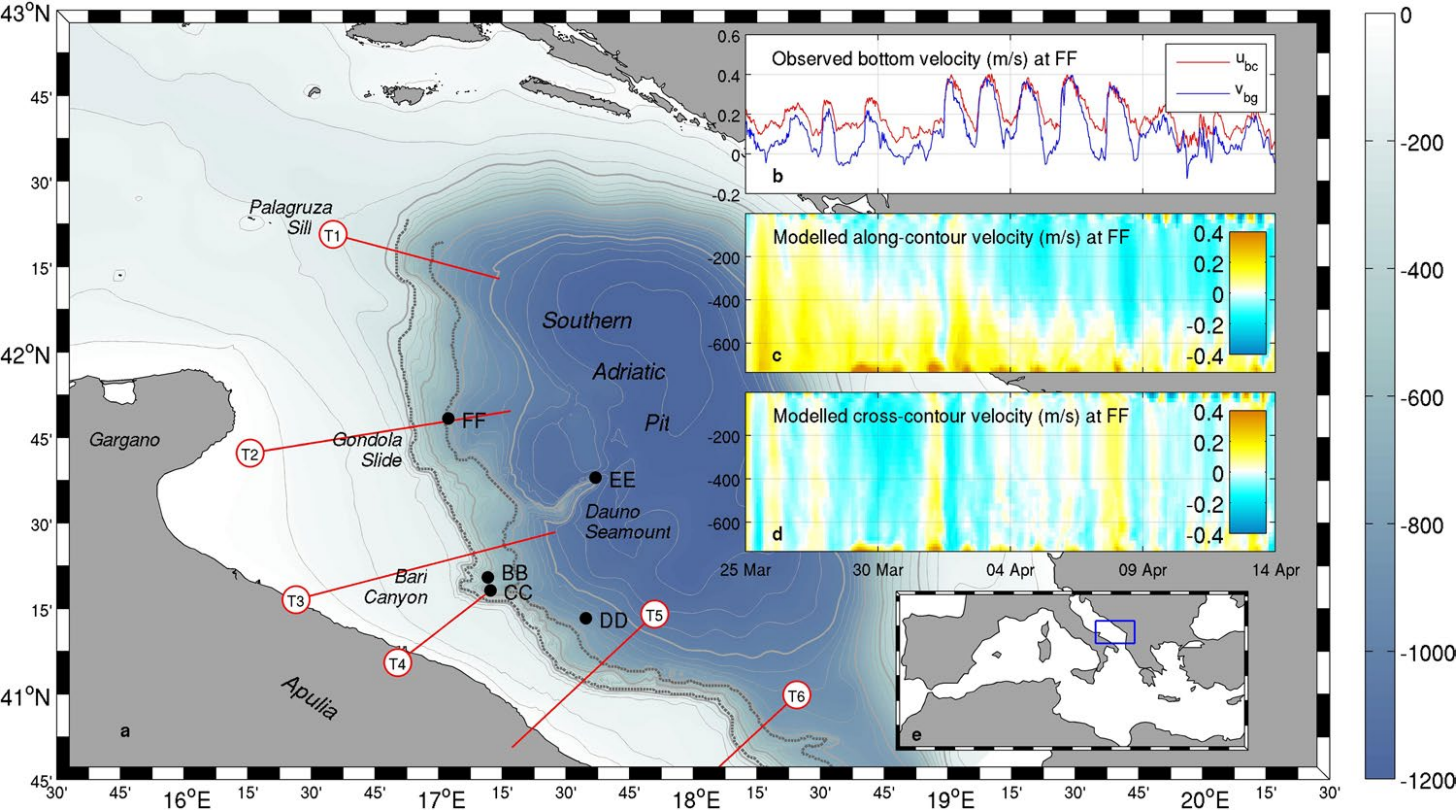
Southern Adriatic bathymetry (**a**) and its position in the Mediterranean basin (**e**). Thick (thin) light grey contours represent 250- (50-) m spaced isobaths, while dashed dark grey lines represent the contour portions considered for the analysis of wave propagation. Red lines depict the transects considered for the analysis of the cross-shelf structure of the signal. Insets show the along-contour (*u*_*bc*_, positive southwards) and cross-contour (*v*_*bg*_, positive off-shelf) components of the observed near-bottom current velocity at FF mooring site (**b**) and the vertical structure of modelled along- (**c**) and cross-contour (**d**) velocity components. Maps were generated by using MATLAB R2012a.

In this study we focus on a particular kind of topographic waves propagating along the continental margin and trapped thereby as an effect of the pronounced bathymetry, usually referred to as Continental Shelf Waves^11–14^ (CSWs), in the southern Adriatic Sea (Fig. 1a,e). The Adriatic Sea is an epicontinental basin of the eastern Mediterranean Sea acting as a “cold engine” for Mediterranean thermohaline circulation, due to the formation of dense water masses (traditionally labelled as North Adriatic Dense Water - NAdDW) through cooling and evaporation in winter^15,16^. The possible occurrence of CSWs on the Southern Adriatic Margin (SAM) was theoretically postulated back in 1990^17^, but up to now never experimentally demonstrated.

An opportunity to tackle this challenge is provided by the dense water event that took place in the Adriatic basin in the 2012 winter-spring period. After an autumn season characterised by small freshwater supply into the northern Adriatic^18^ basin, a cold air outbreak of exceptional intensity hit this area^19–21^, inducing cold and dry northeasterly winds unceasingly blowing over the sea surface for more than two weeks (from 25 January to 13 February 2012). This induced a drop in water temperatures and a rise in salinity^22,23^, leading potential density anomaly to locally exceed 30 kg/m^3 ^^24,25^. In the subsequent weeks, newly formed dense water masses migrated southwards along the continental shelf parallel to the Italian coast, partly descending the continental slope off Apulia, with relevant implications for deep sea ventilation^26,27^ and continental margin reshaping^28,29^. The large-scale pathway of dense waters migration across the continental shelf generally takes place nearly parallel to the isobaths, oscillating around a geostrophic equilibrium under the effect of tides and inertial oscillations^24,30^. Flow trajectories and timing strongly depend on a number of factors, such as the kinetic energy injected in the cooling basin during the formation event, the thermohaline properties of the vein, its interactions with large-scale topographic features (such as the Mid-Adriatic depression^15^) and with the surrounding water masses^24,27,29^. NAdDW migration from the cooling basin to the southern Adriatic Sea generally takes several weeks^31,32^. In the 2012 episode in particular, the long cooling event that occurred from late January to mid February led first to a highly energetic phase (Fabruary to early March), with strong currents triggered by the intense momentum injections that took place during the storm, then to a long period of relatively regular flow (mid March to mid May), and eventually to the complete depletion of the northern basin in June^27,29^. Due to the outstanding intensity of the cooling event, a two-leg Rapid Environmental Assessment oceanographic campaign was carried out in March-April 2012 in the southern Adriatic Sea (Operation Dense Water 2012, ODW2012^33^) in order to investigate the thermohaline and dynamical properties of the dense water vein and of the ambient water, while the continental margin was already monitored by five moored instrumental arrays^26^ (Fig. 1).

During the dense water downflow phase, observations of near-bottom current velocity and potential temperature at mooring FF, deployed in the open slope, highlighted the recurring presence of cold energetic pulses with period between 1.5 and 2.0 days^33^. This signal was found both in the observations and in the numerical model fields^27^, but its origin and drivers have not been explored in detail, notwithstanding the primary role that such relatively high-frequency oscillations may play in cross-shelf transport and horizontal mixing^10^.

In this work we provide a dynamical characterisation of these pulsing features and their implications for continental margin processes and regional circulation. To this aim, we integrate the information provided by mooring records with three-dimensional hydrodynamic fields resulting from a numerical modelling experiment set up for the winter 2012 event and described in previous studies^24,27,29^. In the considered simulation, the whole dense water formation and migration event was reproduced with a 1–km horizontal resolution using the Coupled Ocean-Atmosphere-Waves-Sediment Transport (COAWST) modelling system^34^. Most of the evaluations presented here focus on the period from 25 March to 15 April 2012, when the mooring record is available and the signal is sufficiently steady with a clear modulation signature. Nevertheless, a wavelet analysis on numerical model results carried out throughout the whole simulation period will also be discussed, allowing to appreciate the persistence of this process and its profound connection with dense water migration.

## Results

The components of near-bottom current velocities observed at FF from 25 March to 15 April 2012 (Fig. 1b, water depth 733 m) oscillate between 0.1 and 0.4 m/s southwards in the along-isobath direction and between 0 and 0.4 m/s in the off-shelf direction, with current speed peaks exceeding 0.5 m/s^27^. With reference to the same site, modelled vertical profiles of the velocity components along the same directions (Fig. 1c,d) show that this behaviour, although amplified close to the bottom, exhibits a remarkable vertical extent. This is particularly evident in the cross-contour direction, in which patterns of vertical bands show the periodic modulation of on- and off-shelf transport involving the whole water column.

**Figure 2:**
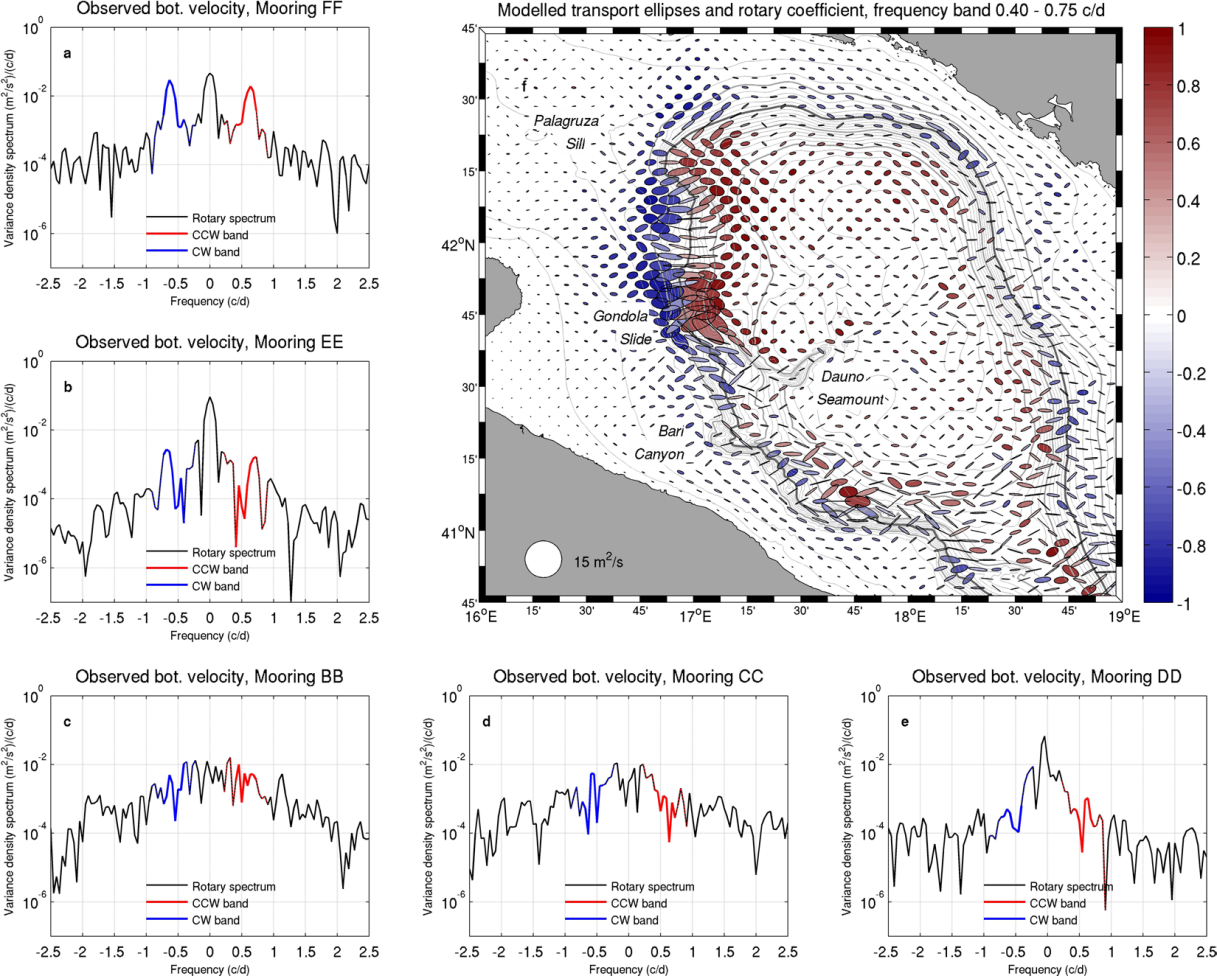
Results of the rotary analysis of observed near-bottom velocity at the mooring sites and modelled horizontal transport in the southern Adriatic basin, 25 March to 15 April 2012. The blue and red solid segments in the rotary spectra (panels a–e) highlight respectively the clockwise and counterclockwise components in the frequency band considered in the analysis, namely 0.4–0.75 c/d (period from 1.3 to 2.5 days). Dashed coloured segments depict the actual span of the buffer frequency bands considered in the filtering algorithm (0.2 to 0.95 c/d). Panel f depicts the average ellipses and rotary coefficients (negative and positive values indicate clockwise and counterclockwise rotation respectively) within the considered frequency band, with modelled transports subsampled every 5 grid points (approximately 5 km) on either direction. The dark grey line indicates the position of the 500 m isobath mentioned in the text; map generated by using MATLAB R2012a.

Measurements from other locations along the continental margin provide a first feeling of the spatial dimension of the dynamics responsible for the observed pulsing signal. Rotary spectra of observed near-bottom velocity computed on each mooring site (Fig. 2a–e) suggest that, besides the evident case of FF, maxima in power spectral density in the frequency band 0.4–0.75 c/d (equivalent to period ranging between 1.3 and 2.5 days) appear with a consistent spatial distribution throughout the western continental margin. Indeed, power spectral density for this frequency band is the largest and most peaked in the northern open slope (FF) and retains relatively high values in the Bari Canyon (BB and CC, water depth 606 m and 595 m respectively), though with less peaked attitude, being in turn slightly weaker but more peaked (with some contribution from higher frequencies) at the tip of the Dauno Seamount (EE, water depth 1194 m). Finally, in the lower slope southeast of the Bari Canyon (DD, water depth 860 m) the signal is largely suppressed.

A dedicated analysis on modelled fields provides a more complete view on the spatial patterns of the observed pulsing features in the SAM. Besides addressing the columnar tendency of the process as highlighted in Fig. 1c,d, the focus on horizontal transport allows to analyse the barotropic effects associated with these pulses (this working hypothesis will be verified in the discussion). The rotary ellipses computed for this vector quantity^35^ (Fig. 2f) within the frequency band under consideration show that the pulses are concentrated on the continental slope, particularly on the western side, generally with a significant cross-shelf component. A preferential clockwise signal is widespread along most of the western sectors of the upper slope, with stronger intensity between Palagruza Sill and the Dauno Seamount. Along this region, such an intense clockwise rotation is associated with a counterclockwise modulation spanning the lower slope, with a net separation in the proximity of the 500 m isobath. The horizontal structure of the pattern is locally interrupted by the Dauno Seamount, which marks the morphology of this sector of the slope and conveys part of the flow towards the center of the pit, and by the Bari Canyon, indenting the continental shelf and the upper slope. South of the Bari Canyon, contour-parallel bands of ellipses with alternate rotation directions partially reappear, though with smaller intensity and spatial consistency, affecting a large region of the southern slope. This picture suggests the presence of a coherent oscillatory feature confined to the continental margin, providing a first element in the direction of interpreting the observed pulses as the signature of the passage of a train of CSWs.

**Figure 3:**
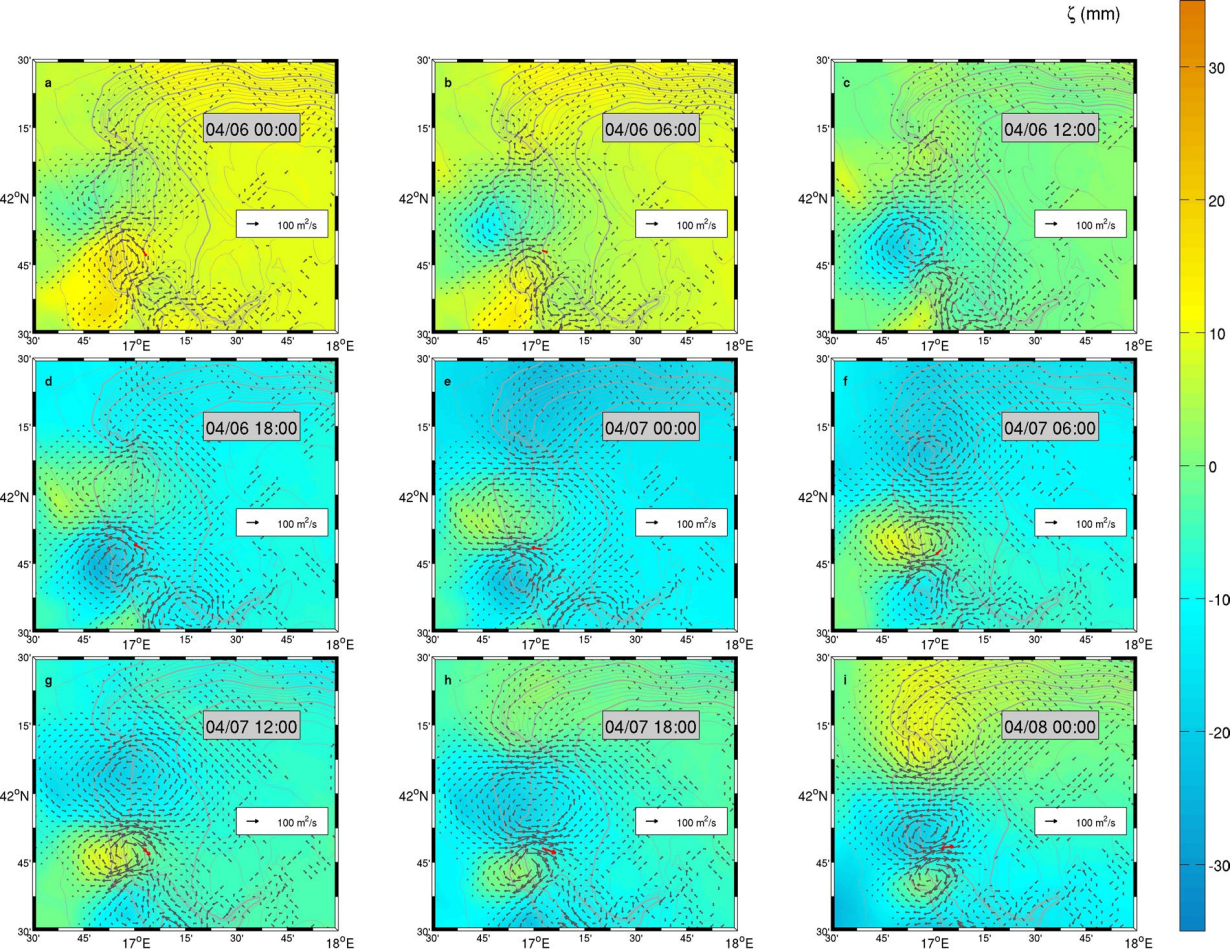
Band-filtered sea surface elevation (colour) and transport (arrows) patterns along the northern sectors of the SAM, depicted every 6 hours from 6 April 2012, 00:00 to 8 April 2012, 00:00. For graphical purposes, transport has been subsampled every 3 grid points and vectors with intensity below 5 m^2^/s have not been represented. The red arrow in each panel indicates transport intensity and direction at FF. Maps were generated by using MATLAB R2012a.

The patterns of band-filtered modelled transport and sea surface elevation signals allow a deeper insight into the spatial properties and the propagation dynamics of the observed oscillations. An overall view of the process evolution during the time span in which the pulses were evidenced in FF mooring data (Fig. 3) highlights the southward propagation of stable circulation cells associated with an along-slope modulation of sea surface elevation, superimposed to a larger-scale component of background oscillation affecting most of the region. Since the algorithm applied to the considered frequency band filters out both the semidiurnal and diurnal tidal components as well as the fundamental Adriatic seiche (with a period of approximately 22 h), such a background modulation (whose amplitude is nonetheless on the order of a few centimetres) is most likely ascribable to the band-passed fraction of the meteorologically-driven oscillations. At the interface between adjacent circulation cells, sea surface elevation is close to the background value and transport intensity is locally maximum, with alternating off- and on-shelf jets yielding up to 100 m^2^/s. Besides, a focus on the transport evolution at a single point (e.g. at FF, red arrow in Fig. 3) throughout subsequent snapshots allows to visualise the vector rotation, supporting the interpretation of Fig. 2f by linking the local value of the rotary coefficient with the migration of the circulation patterns. The net separation between clockwise and counterclockwise transport ellipses in the northwestern slope can be recognised as a signature of a preferential trajectory of the transport cell centroid, while the fragmentation of the ellipse pattern south of the Dauno Seamount can be interpreted as an along-slope modification of the transport structure.

**Figure 4:**
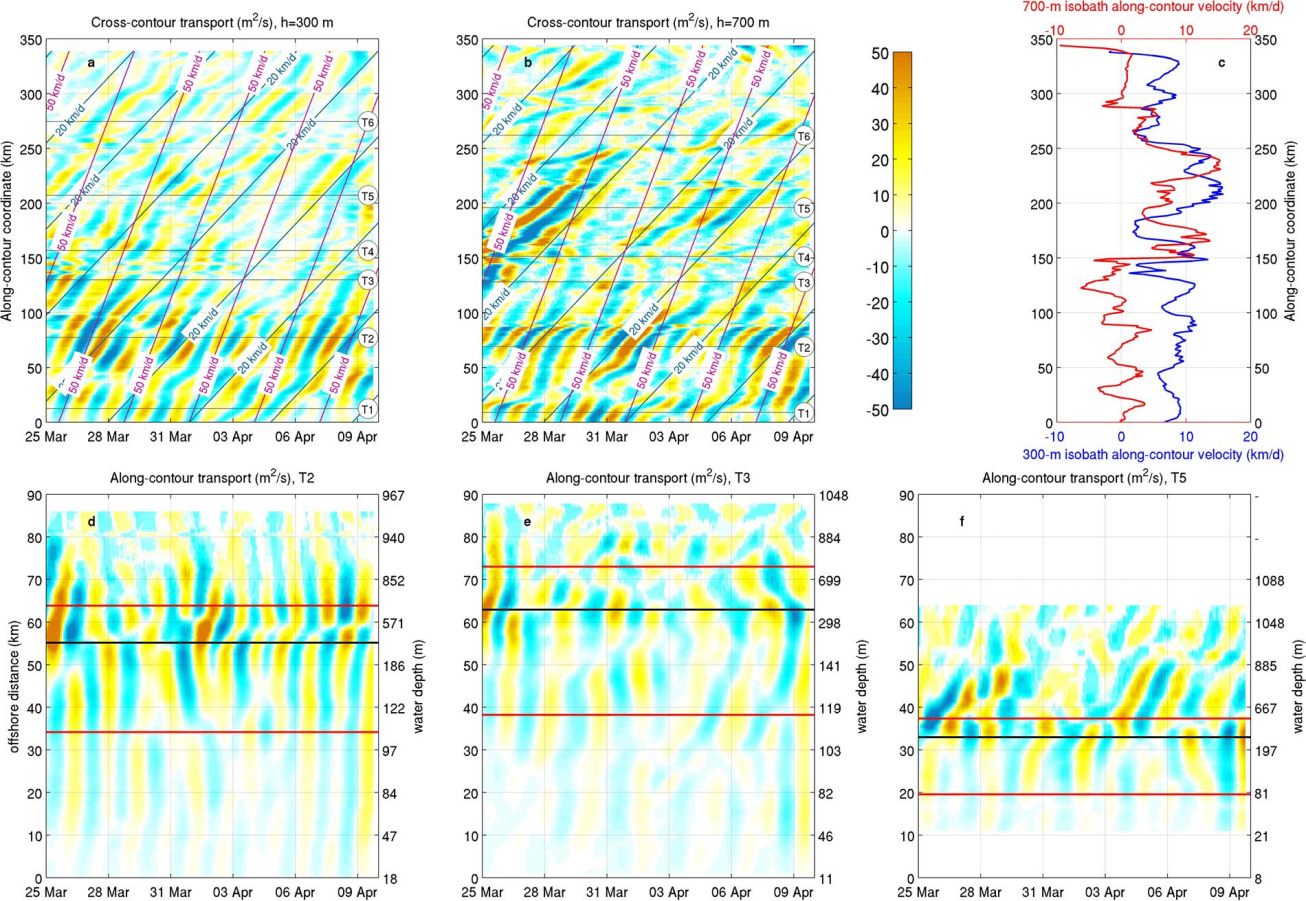
Cross-contour (positive off-shelf) and along-contour (positive southwards) transport time series across the 300 m and 700 m isobath (**a**,**b**) and transects T2, T3 and T5 (**d–f**) respectively. Panel (**c**) shows the distribution of the mean vertically-averaged along-contour velocity *u*_*c*_. Blue and magenta lines on panels a,b represent the slope of transport bands corresponding to circulation cells propagating with 20 km/d and 50 km/d along-contour speed, respectively. The theoretical position of zero-crossings in the instantaneous distribution of along-contour transport across the continental slope (computed by setting *q*_*c*_ = 0 in Eq. 1) is represented in panels d–f by black and red horizontal lines, with reference to oscillation modes 1 and 2 respectively.

After having characterised the regional circulation features associated with the observed near-bottom velocity pulses, their explanation in terms of CSWs requires a verification of the compatibility of the modelled wave parameters against the theoretical dispersion relation for these perturbations. In this direction, a quantitative evaluation of wavelength, oscillation modes, and phase speed along the SAM can be obtained by considering the time series of transport signals related to band-filtered velocity fields along selected isobath contours and transects. The space-time representation of cross-shelf transport evolution along the contours (Fig. 4a,b) allows to assess the spatial coherence and the migration dynamics of the patterns. In particular, darker regions identify maxima in off-shelf (positive) and on-shelf (negative) transport intensity, and the bands in the diagrams track the migration of the cross-shelf jets delimitating the circulation cells. The apparent propagation speed *v*_*app*_ of the waves can therefore be estimated from the slope of the transport bands. This highlights the presence of coherent structures propagating at slightly less than 50 km/d along the northern part of the continental slope, dropping to smaller speed values south of the Bari Canyon (T4). The apparent speed for these structures, mostly spanning a 20–50 km/d variability range, allows to estimate their wavelength *λ* as the ratio between *v*_*app*_ and the observed frequency $\bar{{\omega }}$. Since in this case $\bar{{\omega }}$ is the average of the considered frequency band, namely 0.575 c/d (corresponding to a 42-hour period), the wavelength of the structures varies between 35 and 87 km. The background along-contour current *u*_*c*_, to be considered for the correction of the Doppler effect modulating the signal frequency^13^, is on the order of 10 km/d along most of the 300 m isobath, while it oscillates around zero along the northwestern sector of the 700 m isobath and around 10 km/d south of the Dauno Seamount (Fig. 4c).

The wave modes can be estimated from the evaluation of the number of zero-crossings in along-contour transport patterns across the continental slope on selected transects, each representative of a different sector of the margin (Fig. 4d–f). The generic *k*−th mode is indeed associated with a cross-slope structure of the along-contour transport *q*_*c*_ that, for relatively short waves, is given by

1 $${q}_{c}\propto b{e}^{b(y-L)}\,\sin \,(\frac{k\pi y}{L})+\frac{k\pi }{L}{e}^{b(y-L)}\,\cos \,(\frac{k\pi y}{L})$$

where *y* is the cross-slope coordinate (positive offshore), *b* is the slope steepness, and *L* is the slope width. This means that, as far as the transects are actually perpendicular to the isobaths, a mode *k* wave will be characterised by *k* zero-crossings along the slope, that will in turn indicate the passage of as many circulation cells. It is worth noting that a local deviation from orthogonality, as well as the presence of residual noise or other signals in the filtered time series, can hamper this assessment, whose validity needs again to be confirmed by comparison with the dispersion relations. Thus, in the northwestern sectors of the slope (T2), it is possible to observe a stable mode 1 wave propagation, while between the Dauno Seamount and the Bari Canyon (T3) mode 1 oscillations seem to coexist with other oscillation components. In both T2 and T3 the position of the zero crossing in the transport distribution fits well with the value prescribed by the theoretical formulation. South of the Bari Canyon (T5) the pattern is significantly less organised, with mostly relatively weak mode 2 perturbations taking place, with a deviation from the theoretical localisation of the zero crossing on the order of 10 km.

**Figure 5:**
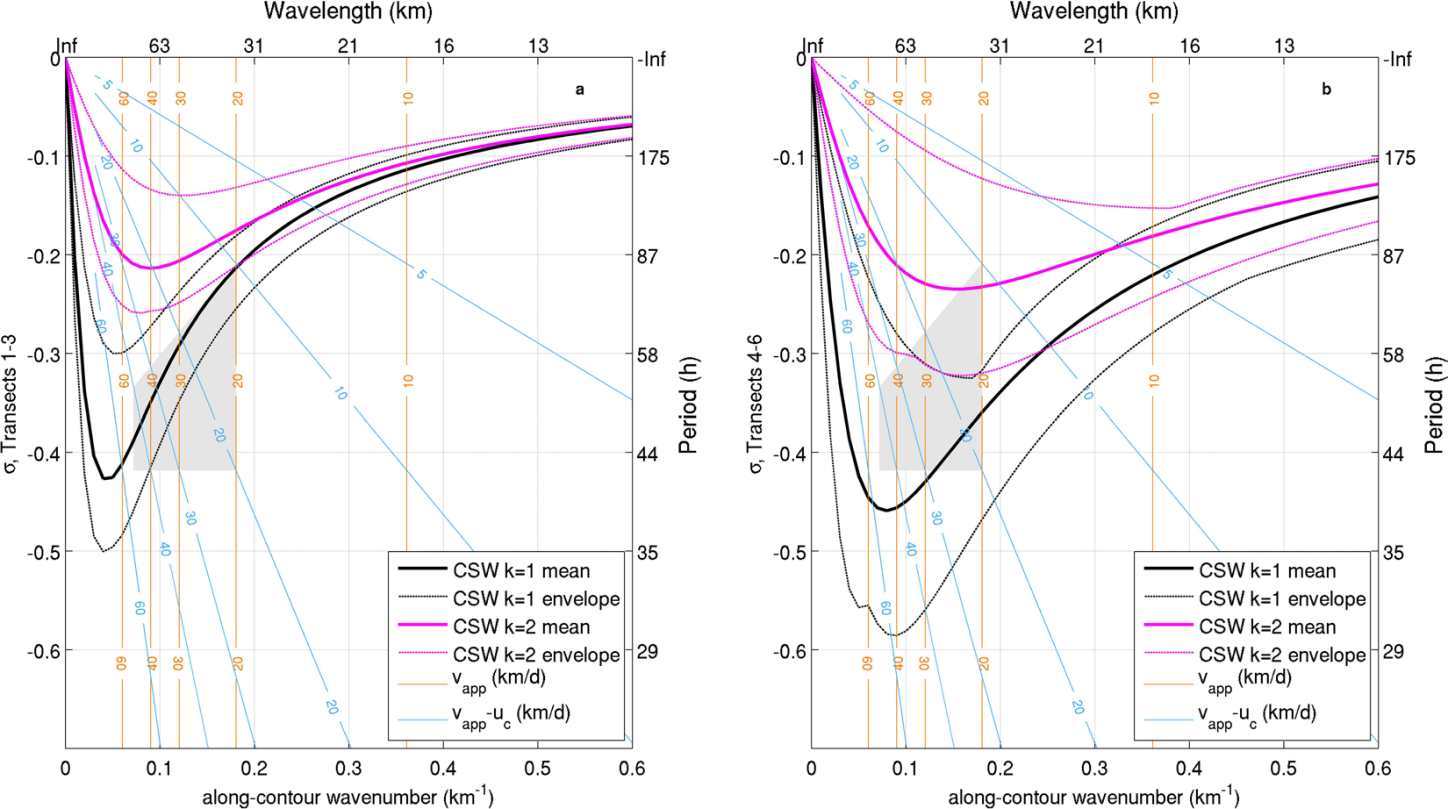
Means (thick lines) and envelopes (dashed lines) of the theoretical mode 1 (black) and mode 2 (magenta) CSW dispersion relations associated with continental margin morphology north (**a**) and south (**b**) of the Bari Canyon; *σ* represents the frequency normalized with respect to the Coriolis parameter. Orange lines represent the relationship between apparent propagation speed *v*_*app*_ and wavelength *λ* for the considered frequency band (0.4–0.75 c/d, with mean value $\bar{{\omega }}$ = 0.575 c/d). Light blue lines represent the Doppler correction for determining the real frequency *ω* of a signal with observed frequency $\bar{{\omega }}$, propagating in a medium with a background current *u*_*c*_ (positive along the wave propagation direction), namely $\omega =\bar{{\omega }}\frac{{v}_{app}-{u}_{c}}{{v}_{app}}$. The intersections between orange and light blue lines thus identify the wavelengths and Doppler-corrected frequencies for given apparent propagation speeds and background currents. The grey shaded area encloses the region representing most of the signal variability depicted in Fig. 4.

With these elements it is now possible to verify that the observed features are compatible with a CSW train propagating along the SAM. Figure 5 represents the envelopes of mode 1 and mode 2 dispersion relations for CSWs associated with the continental margin morphology north (panel a) and south (panel b) of the Bari Canyon. The amplitude of the envelopes reflects the variability in the continental margin morphology, computed as an exponential fit along seven transects (not all shown here) along each tract. Superimposed on the dispersion relation curves, sets of straight lines allow to locate in the diagram the positions of perturbations identified in Fig. 4, characterised by given values of *v*_*app*_ (orange lines in Fig. 5) in the presence of a background current with *u*_*c*_ along-contour velocity (blue lines). In particular, while orange lines depict the correspondence between *λ* and *v*_*app*_, blue lines represent the values of the corrected frequency *ω* of a signal with observed frequency $\bar{{\omega }}$ as a function of the apparent speed *v*_*app*_ for different values of the background current speed, following the relation for the Doppler shift: $\omega =\bar{{\omega }}\frac{{v}_{app}-{u}_{c}}{{v}_{app}}$. Within this scheme, the gray shaded polygon represents the region encompassing the wave parameters of the features detected in Fig. 4, with wavelength between 35 and 87 km and background current on average between 0 and 10 km/d. In particular, the signal between transects T1 and T3 at 300 m depth can be localised towards the relatively low-frequency, large-wavelength region of the quadrilateral (${v}_{app}$  ∼50 km/d, Doppler shifted by approximately 10 km/d), whereas the pattern associated with the same sector at 700 m depth mostly lies close to the high-frequency side (*v*_*app*_ 20–50 km/d, *u*_*c*_ close to zero). In turn, the patterns depicted between T4 and T6 tend to spread along the low-frequency side of the polygon (*v*_*app*_ 20–50 km/d, *u*_*c*_ around 10 km/d). These values, associated with a corrected period ranging between 2 and 4 days, are compatible with the dispersion relations for the first two CSW modes allowed by the western SAM morphology^11^, with a dominance of mode 1 in the northern sectors and of mode 2 south of the Dauno Seamount.

Besides confirming the occurrence of CSWs in the southern Adriatic Sea, the emerging picture provides an insight into the propagation of these waves and their modification as a response to along-contour variations in continental margin morphology^36,37^. Since the lower modes are generally most excitable^38^, it is not surprising to find mode 1 waves along the open slope downstream of the generation zone, at least as long as the seabed topography is sufficiently regular. Changes in the oscillation mode and in the energy content (visible in the rotary coefficient patterns and ellipse amplitude in Fig. 2f) occur indeed when the CSW train crosses the Dauno Seamount region, thus subsequently passing a widening and a narrowing of the continental margin. In particular, a widening cross-shelf profile fosters the scattering of the incident signal into higher modes, that increase their relative weight as the variation becomes larger^12,38,39^, generally with little or no energy reflection^40^. By contrast, profile narrowing tends to partially prevent energy propagation by favouring backscattering and local interference between incident and reflected waves. Scattering provided by bathymetric irregularities is also associated with energy damping, which is stronger when the scattered modes have group speed close to zero, and variations in wave phase speed^36^. A further look at Fig. 5 suggests that this is the case south of the Bari Canyon, where most of mode 1 incident waves are scattered into mode 2 waves close to the extreme of the dispersion relation (where the group speed, equal to ∂*ω*/∂*γ*, is very small), while only shorter mode 2 waves have positive group speed. Besides inducing a Doppler shift on the signal, the presence of a background along-contour flow is expected to concentrate the scattering within a limited number of relatively low, forward-propagating modes, tendentially preventing backscattering and favouring the activation of evanescent modes^13^. In turn, although the presence of a velocity shear can in principle trigger additional oscillation modes^41^, the persistent dominance of mode 1 north of the Dauno Seamount shows that this was not the case in the considered event.

**Figure 6:**
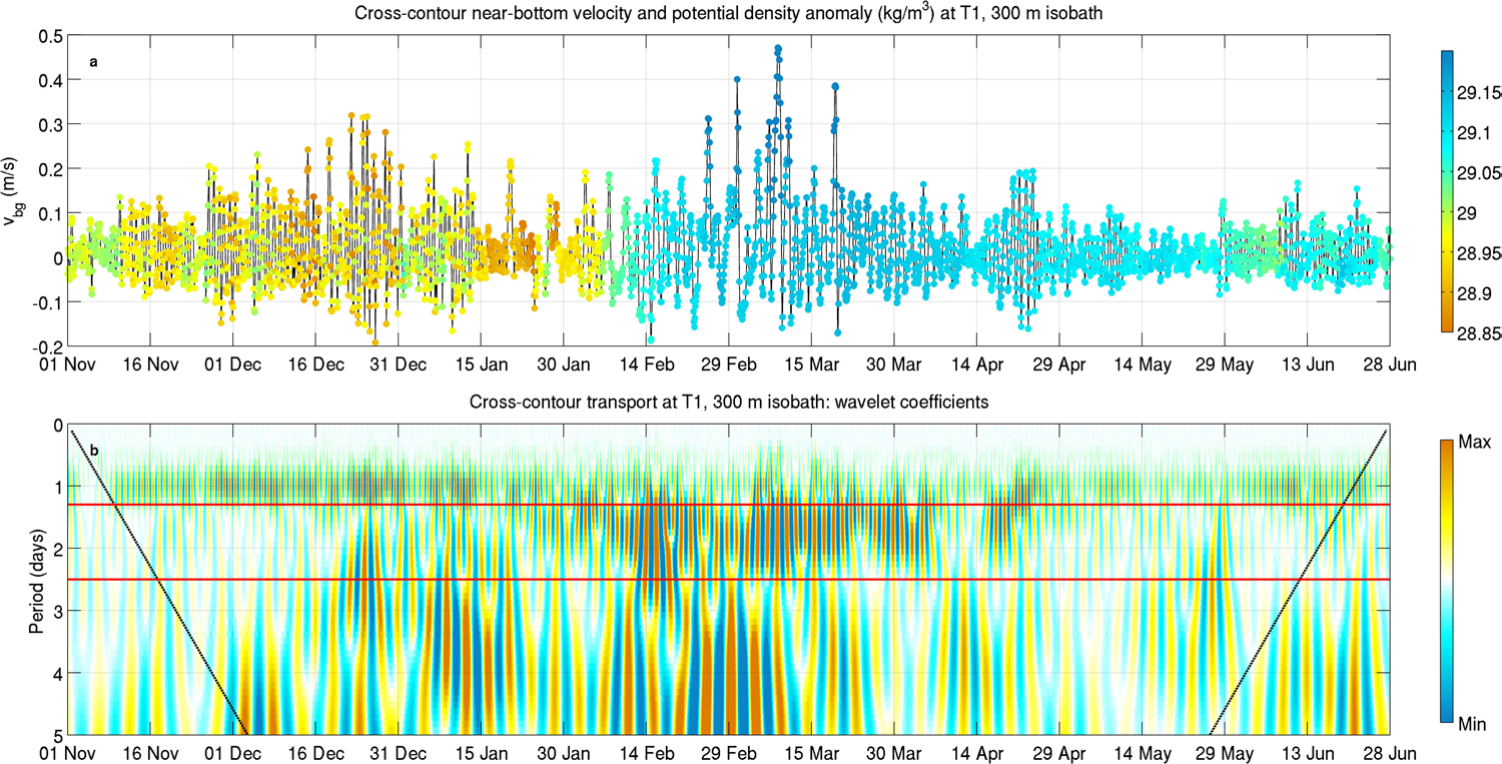
Time series of cross-contour near-bottom velocity and potential density anomaly (**a**) and wavelet coefficients of cross-contour transport (**b**) at T1, 300 m isobath. Dashed black lines close to either end of the plot in panel b indicate the cone of influence in which edge effects can be important, while red thick lines delimitate period values ranging between 1.3 and 2.5 days.

The identification of the originating mechanisms and the temporal extent of CSW generation in the SAM requires to consider near-bottom current dynamics at the northern edge of the continental margin during the whole period encompassed by the numerical model run^24^. Wavelet analysis presented in Fig. 6 shows that cross-contour transport pulses with period between 1.3 and 2.5 days (corresponding to the frequency band of the pulses observed in the mooring signals and here interpreted as CSWs) appear in concurrence with the influx of dense water masses following NAdDW formation in the northern basin^24^, persisting for several weeks during the most intense phase of the process^27^, from February to April 2012. The localised appearance of energetic, strongly ageostrophic currents associated with dense water downflow across the northernmost sectors of the SAM, also testified by geological evidences^28,29^, thus seems to trigger a perturbation in the potential vorticity field, capable of subsequently propagating along the slope as a CSW train.

## Discussion

Based on the analysis of mooring data and high-resolution hydrodynamic model fields it was possible to provide a physical interpretation of the modulation observed in near-bottom velocity and thermohaline quantities during a dense water downflow event on the SAM^33^, thus demonstrating for the first time the occurrence of CSWs in this area. The observed pulses are indeed associated with a train of CSWs generated on the northern sector of the shelf break, having a 2–4 day effective period and 35–87 km wavelength, and propagating southwards trapped over the continental slope at a depth approximately varying from 250 to 1000 m. Wave propagation speed ranges between 20 and 50 km/d, and is increased by an average background along-contour current up to 10 km/d, which Doppler-shifts the frequencies to the values observed in mooring data. The oscillation, triggered by ageostrophic dense water inflow across the northernmost sectors of the SAM, is dominated by mode 1 north of the Dauno Seamount. Topographic effects in the Dauno region induce energy scattering and damping in the propagating waves, inducing a transition towards mode 2 dominance in the southwestern quadrant of the margin, with a significantly smaller energy content.

In this analysis the exponential approximation of the off-shore continental margin profiles provided, in the simplest available formulation, a consistent theoretical framework for the oscillations observed in mooring data records and reconstructed from the numerical model fields and for the main features of their along-slope variations. The description of additional effects related to small-scale geomorphological elements or local discontinuities in the margin topography is beyond the scope of the present work, but it can be explored in upcoming studies based on higher-detail representations of the seabed morphology^14,42^, though likely at the price of some loss of immediacy in the interpretation of the results.

Albeit the process is related to a dense water downflow process, the barotropic approach is sufficient to provide a dynamical interpretation of the pulses under examination with the manageability of an analytical formulation, not available for the baroclinic case^43^. Computing the Burger number *Bu* = *NH*/*fL* (where *N* is the Brunt-Väisälä frequency, *H* and *L* are the vertical and horizontal scales of the motion respectively, and *f* is the Coriolis frequency) by substituting the typical values for the 500 m isobath, values generally smaller than 0.3 are obtained, providing further support to our assumption.

A major implication of the present results is that dense water downflow along the Adriatic continental margin is not only driven into preferential pathways locally identified by topographic singularities, but can also be triggered or significantly influenced by an incident CSW train generated tens to hundreds of kilometres upstream. The area involved by upwelling and downwelling fluxes is thus remarkably larger than in the case of a local topographic driver (e.g., a submarine canyon), encompassing wide sectors of the open slope.

The significance of this teleconnection structure, which has several traits in common with other cases throughout the world ocean (for instance, the flow pattern induced by topographic waves in the Great South Australian Coastal Upwelling System^9,44^), should be explored in the broader framework of the quantification of heat, carbon, oxygen and sediment exchanges between the continental shelf and the abyssal regions. In an ecological perspective, this kind of information can help characterising the functioning of high-productivity ecosystem cells^45,46^, addressing some of the open questions concerning the relationship among hydrodynamics, migration routes between abyssal and coastal regions, and the biological cycles of some marine species^47^. A possible immediate outcome of the new insight on NAdDW dynamics made available by this work is a deeper comprehension of the role of dense water in controlling cold water coral distribution along the SAM^48^ as well as in other marginal systems^49,50^, towards a numerical model-based multidisciplinary characterisation of deep sea habitats^51^.

## Methods

### Mooring data

Mooring data used in this work are provided by Aanderaa RCM7 and RCM8 current meters deployed along different sectors of the SAM. Each of the five deployment locations has been chosen based on geomorphological evidences and preliminary model estimates of likely preferential dense water pathways^33^. Single-point current data were sampled with 30 min interval at 8.5 m above the sea bottom. Available data cover the period from 8–9 March to 12 June 2012. For further details on the mooring arrays the reader is referred to the relevant publications^26,27^.

### Numerical model

Hydrodynamic fields were obtained for the period from November 2011 to June 2012 by a wave-ocean currents simulation based on a two-way coupled implementation^24^ of SWAN (Simulating WAves Nearshore)^52^ and ROMS (Regional Ocean Modeling System)^53^ models within the COAWST system^34^ over a domain with 1 km horizontal resolution, vertically discretised into 30 terrain-following sigma levels. Hourly atmospheric forcings were provided with 7 km horizontal resolution by the operational COSMO-I7 model run by the Emilia Romagna Region Meteorological and Hydrological Service (ARPA-EMR-SIMC Bologna, Italy; http://www.arpa.emr.it/sim/pagine/home/index). Daily fields of sea surface elevation, momentum, temperature and salinity were provided with 1/16 horizontal resolution at the southern boundary (Otranto Strait) from the Mediterranean Forecasting System^54^ (MFS). At the same boundary, eight tidal constituents (M2, S2, N2, K2, O1, K1, P1, Q1) were also imposed from the Oregon State University Tidal model^55^. A thorough validation of the model run was described in previous works^24,27^, highlighting a satisfactory agreement with observed processes notwithstanding a bias in salinity and potential temperature leading to a generalised 0.25 kg/m^3^ overestimate in potential density. Consistently with the procedure followed and broadly discussed in those works, the estimate of potential density anomaly and the identification of water mass properties were here referred to the unbiased values of potential density. The model validation^27^ against velocity profiles retrieved from L-ADCP casts in the southern Adriatic Sea has shown a −0.01 m/s bias in current speed and a −46° bias in direction, while the comparison against near-bottom current time series at the mooring sites provided a similar result in terms of current speed (−0.02 m/s bias), but far better performances in terms of direction (bias smaller than 1°). Additional information on the model implementation, as well as extensive discussions and statistics on the model performance can be found in dedicated papers^23,24,27^.

### Rotary analysis and signal filtering

Rotary analysis^35^ allowed to compute the counterclockwise and clockwise (*S*^+^ and *S*^−^ respectively) components of the spectrum as a function of the autospectra *S*_*uu*_ and *S*_*vv*_ and quadrature spectra *Q*_*uv*_ of the velocity components:

2 $${S}^{+}={S}_{uu}+{S}_{vv}+2{Q}_{uv}\,\,\,\,\,\,{S}^{-}={S}_{uu}+{S}_{vv}-2{Q}_{uv}$$

The rotary coefficient *r*, indicating a counterclockwise (if positive) or clockwise rotation (if negative), has been computed as

3 $$r=\frac{{S}^{+}-{S}^{-}}{{S}^{+}+{S}^{-}}$$

The major and minor axes *l*_*maj*,*min*_ of the rotary ellipses and their orientation *α* with respect to the zonal direction, *S*_*uv*_ being the cospectrum of the velocity components, have been calculated as:

4 $${l}_{maj,min}=\sqrt{{S}^{+}}\pm \sqrt{{S}^{-}}$$

5 $$\alpha =\frac{1}{2}{\tan }^{-1}(\frac{2{S}_{uv}}{{S}_{uu}-{S}_{vv}})$$

The time series of the vertically-averaged zonal and meridional velocity components in each grid point were filtered by means of a Parks-McClellan algorithm embedded in MATLAB, providing full amplitude response in the 0.4–0.75 c/d frequency band, decreasing to zero in the buffer bracketed within 0.2 to 0.95 c/d. The inclusion of a buffer in the filtering band is the result of the trade-off between the order of the filter (reflecting on its capability of approximating the desired amplitude response) and the number of samples available for reconstructing the filtered time series, since the application of a *n*−th order filter comes at the cost of losing *n*/2 samples from the reconstructed time series.

### Dispersion relation computation

**Figure 7:**
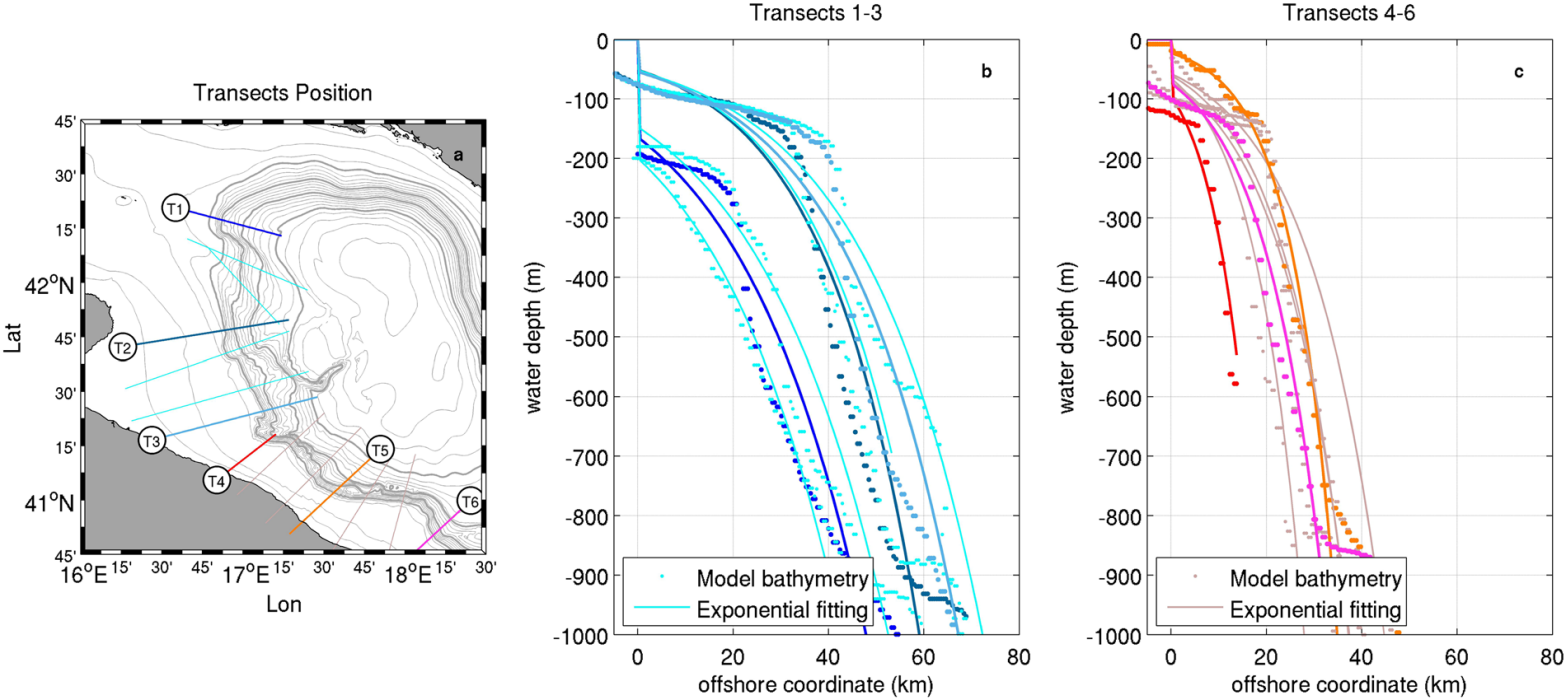
Position of the transects used in the characterisation of the continental margin morphology (**a**, with thick and thin light grey contours representing 250- and 50- m spaced isobaths respectively) and comparison between the realistic bathymetry ingested in the numerical model and the idealised fitting in the exponential form described in Eq. 6, north (**b**) and south (**c**) of the Bari Canyon. Map generated by using MATLAB R2012a.

Theoretical dispersion relations for CSWs^11^ were identified considering a continental margin bordering a flat-bottomed ocean, whose typical idealised transect geometry can be described in the exponential form

6 $$h(y)={h}_{0}{{\rm{e}}}^{2by}\,\,\,\,\,\,0\le y\le L$$

where *h*_0_ is the onshore depth of the continental shelf, *b* is the parameter accounting for the slope steepness, and *L* is the width of the continental margin along the off-shelf distance *y*. Although not representing local topographic singularities, this approximation (see Fig. 7) allows to capture the main features of the margin geometry as well as their spatial variability, with values of *b* and *L* respectively oscillating around 2.2e-5 m^−1^ and 50 km north of the Bari Canyon and around 4.4e-5 m^−1^ and 35 km in the southern tract.

In this configuration, the dispersion relation for a wave propagating along the shelf is given by

7 $$\sigma =-\frac{2b\gamma }{{m}^{2}+{b}^{2}+{\gamma }^{2}}$$

where *σ* is the angular frequency normalised by the Coriolis parameter, *m* is the cross-shelf wavenumber, and *γ* is the along-shelf wavenumber. The application of the boundary conditions leads to a further equation allowing to close the problem, namely

8 $$\tan (mL)=-\frac{m}{|\gamma |+b}$$

Indeed, by solving Eq. 8 for variable *γ* and for the different intervals in which tan(*mL*) is defined and continuous, it is possible to obtain the values of *m* to be used in Eq. 7 for different oscillation modes, allowing to plot the dispersion diagram for a given transect.This procedure was applied to all the transects considered along the SAM, by first computing the geometrical coefficients *h*_0_ and *b* via least-square fitting, and by then numerically solving Eq. 8. In order to account for the variability of the continental margin geometry, we focused our analysis (and the comparison with the modelled waves features) on the envelopes of the dispersion curves computed along different transects within two tracts (7 transects, not all shown in Fig. 1, for each tract) of the continental margin, north and south of the Bari Canyon.

### Data availability

The datasets generated and/or analysed during the current study are available from the corresponding author on reasonable request.
